# The Impact of Heli-FX EndoAnchor Application on Endograft Material: An Experimental Study

**DOI:** 10.1016/j.ejvsvf.2024.09.001

**Published:** 2024-09-10

**Authors:** Damir Vakhitov, Nabil Chakfé, Frédéric Heim, Arindam Chaudhuri

**Affiliations:** a GEPROMED, Strasbourg, France; b Vascular Centre, Tampere University Hospital and Tampere University, Tampere, Finland; c The Department of Vascular Surgery, Kidney Transplantation and Innovation, Strasbourg University Hospital, Strasbourg, France; d Laboratoire de Physique et Mécanique Textile, Université de Haute-Alsace, Mulhouse, France; e Bedfordshire - Milton Keynes Vascular Centre, Bedfordshire Hospitals NHS Foundation Trust, Bedford, UK

**Keywords:** Aortic endoprostheses, Degradation, Expanded polytetrafluoroethylene, Polyester

## Abstract

**Objective:**

The physical impact of the application of Heli-FX EndoAnchors (EA; Medtronic, Minneapolis, USA) on endograft (EG) material is unclear. This study aimed to examine the possible EG membrane alterations after EA implantation.

**Methods:**

Heli-FX EndoAnchors were applied *in vitro* into four aortic endocuffs: AFX2 (Endologix Inc., Irvine, USA); Endurant II (Medtronic, Minneapolis, USA); Gore Excluder (W.L. Gore and Assoc., Flagstaff, USA); and Zenith Renu (Cook Aortic Interventions, Bloomington, USA). Two of these, Endurant II and Renu, are made of polyethylene terephthalate (PET), while Excluder and AFX2 are made of expanded polytetrafluoroethylene (ePTFE). The penetration angle was measured for each EA. The EAs were then carefully removed, and perforations examined with digital and fluorescent microscopy. The area and perimeter of the holes were digitally calculated, and material alterations were analysed.

**Results:**

Of the 13 EAs applied, 12 remained in place. The mean penetration angle was 79°. The ePTFE perforations had oval openings, while PET perforations were round. After EA removal, comparisons between ePTFE and PET material perforations suggested a larger hole area (*p* = 0.011) and perimeter (*p* = 0.003) in the former. The ePTFE perforations in the AFX2 were the largest compared with the holes in other endocuffs (*p* = 0.050). The perforation channel of the ePTFE membrane of the Excluder cuff retained its form after EA removal. Local dissection like layer damage extended further. The perforations in both the Endurant II and the Renu endocuffs shared similar characteristics, with multiple fibres of PET elongated, distorted, or ruptured.

**Conclusion:**

During EA placement, the EG membrane undergoes local alteration and or destruction. Expanded PTFE, particularly AFX2 endocuffs (for which EA use is not recommended), are characterised by a more extensive degree of material alteration compared with PET. Additional studies are required to chronologically supplement these findings in fatigue tests.

## Highlights

• Endoanchors can cause extensive material alteration of expanded polytetrafluoroethylene.
• The EndoAnchor perforations of the Endologix AFX2 are the largest in size.
• The recoil of PET is significant, which may support the EndoAnchor in place.

## INTRODUCTION

Endovascular aneurysm repair (EVAR) is a relatively durable procedure for the treatment of aortic aneurysms.^1^^,^^2^ However, inadequate endograft (EG) proximal sealing to the aortic wall and or neck dilatation^3^ may remain issues, leading to post-operative complications such as EG migration and type I endoleak (T1EL). These high flow endoleaks (EL) are stated to occur in 3–15% of cases.^4^^,^^5^ The condition requires prompt treatment in most cases, as continued T1ELs pressurise the aneurysm and can lead to rupture.^6^^,^^7^

The Heli-FX EndoAnchor (EA) system (Medtronic, Minneapolis, USA) is intended to reduce the risks of migration and ELs by enhancing the durability of an EG sealing to the aortic wall, particularly in the case of challenging anatomy,^8^^,^^9^ aiding stable aortoprosthetic fixation with significant resistance to pullout forces and therefore migration.^10^^,^^11^ The reported data suggest that EA application is a useful adjunct to EVAR, both in managing T1EL,^12^ adding tolerance to aortic neck dilatation,^13^ and aiding post-EVAR sac regression,^8^^,^^14^ leading to the term endosuture aneurysm repair (ESAR).^9^ The technical success rate of the procedure is high at >90%.^15^^,^^16^ Over 90% of patients treated with an EA remain free of type Ia EL (T1aEL).^15^^,^^17^ However, the best results are obtained in prophylactic cases. Conversely, patients treated for T1aEL are still at risk of presenting with the same persistent ELs, which occur in 22.6–34% of cases.^15^^,^^16^ The reasons for failure are insufficiently studied, and available long term evidence is scarce. The European Society for Vascular and Endovascular Surgery currently suggests that further data from scientific studies are needed before EAs can be recommended for routine use in clinical practice.^18^

This experimental series aimed to investigate the qualitative and quantitative impact of EA application on two types of EG material.

## MATERIAL AND METHODS

Endocuffs from four different manufacturers were used to test the influence of Heli-FX EAs on EG material: AFX2 (Endologix Inc., Irvine, USA); Endurant II (Medtronic, Minneapolis, USA); Gore Excluder (W.L. Gore and Assoc., Flagstaff, USA); and Zenith Renu (Cook Aortic Interventions, Bloomington, USA), hereafter referred to as AFX2, Excluder, Endurant II, and Renu. All but one are known to be compatible with the Heli-FX EA system.^19^ Treatment with the Heli-FX EA is contraindicated in conjunction with the Endologix Powerlink.^19^ The AFX(2) is its successor^20^ but the contraindication remains. The EAs (measuring 3 x 4.5 mm) were applied in the usual manner from the inside of the prostheses according to the instructions for use.^19^ The application process imitated real life conditions. All procedures were undertaken by the same experienced vascular surgeon (AC). The EGs were fixed at both sides to keep them in place, and EAs were deployed under precise visual control throughout the procedure.

The endocuff samples with EAs applied within were then analysed in the GEPROMED laboratory. The examination protocol included the following steps, and the same parameters were applied for all studied specimens:

1) Naked eye examinations, followed by imaging of the endoprosthesis samples with a Nikon D5100 camera (Nikon France, Champigny Sur Marne, France). Penetration angles between the longitudinal axes of the EAs and the surface of the endocuffs were measured.
2) Membrane examinations of both sides of the EA application sites with EAs in place were undertaken with a Keyence VHX-600 visible light digital microscope (Keyence France, Courbevoie, France); 5x – 50x magnification was used to analyse the perforated areas. The images were examined, referenced, and stored in a database.
3) Precise removal of the EAs and extensive analysis of the perforation area were undertaken with the same digital microscope. Each visible material defect was examined, referenced, and stored in a database. The area and perimeter of each perforation were measured digitally using dedicated microscope software (Keyence France, Courbevoie, France). Afterwards, the material defects were studied with a Nikon Eclipse Ci (Nikon Corp. Tokyo, Japan) high sensitivity fluorescent microscope with a magnification of 4x/0.013–20x/0.5. The images were stored in a database.

Ethical approval was not required as no patient data or patients were involved.

### Statistical analysis

IBM SPSS Statistics version 29.0 (IBM Corp., Armonk, NY, USA) was used for statistical analysis. Quantitative values were presented as numbers and percentages. Data were tested for normality with the Shapiro–Wilk test. The data that followed normal distribution were presented as mean values with standard deviation (SD) and compared with the independent samples *t* test. Non-normally distributed data were presented as median values with interquartile ranges (IQR) and compared using the Mann–Whitney *U* test. The level of statistical significance was set at <0.050.

## RESULTS

A summary of the studied prostheses and applied EAs is presented in Table 1. Of the 13 EAs applied, 12 remained in place until the visual examination. One EA (4E) disconnected from the cuff during transportation to the laboratory, despite careful shipping in a protection box. Of the four cuffs, two are made of expanded polytetrafluoroethylene (ePTFE; AFX2 and Excluder) and two of polyethylene terephthalate (PET; Endurant II and Renu). The mean penetration angle was 78.9° (SD 9.0°). Of the 13 EAs, three (23.1%) were in contact with the metal stent surface of the endocuff.

**Table 1:** Basic characteristics of the studied aortic cuffs and applied EndoAnchors.

Endocuff producer	Material	EA subgroup number	EA penetration angle (°)	EA contact with stent	Degree of EA penetration
AFX2	ePTFE	1E	90	No	^4^/_4_
AFX2	ePTFE	2E	85	No	^4^/_4_
AFX2	ePTFE	3E	80	No	^3^/_4_
AFX2	ePTFE	4E	n/a	No	^3^/_4_
Excluder	ePTFE	1G	60	No	^4^/_4_
Excluder	ePTFE	2G	87	No	^4^/_4_
Excluder	ePTFE	3G	75	Yes	^3^/_4_
Excluder	ePTFE	4G	85	Yes	^1^/_4_
Endurant II	PET	1M	80	No	^4^/_4_
Endurant II	PET	2M	85	Yes	^3^/_4_
Renu	PET	1C	75	No	^4^/_4_
Renu	PET	2C	65	No	^4^/_4_
Renu	PET	3C	80	No	^1^/_4_

Abbreviations: EA = EndoAnchor; ePTFE = expanded polytetrafluoroethylene; PET = polyethylene terephthalate; EG = endograft; n/a, not applicable); AFX2 (Endologix Inc., Irvine, USA); Gore Excluder (W.L. Gore and Assoc., Flagstaff, USA); Endurant II (Medtronic, Minneapolis, USA); Zenith Renu (Cook Aortic Interventions, Bloomington, USA).

### Digital microscopy

**Figure 1:**
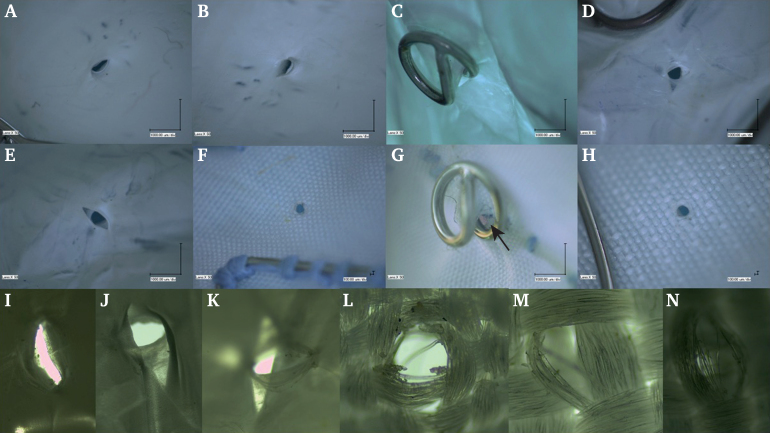
Digital and fluorescent microscopy examples of perforations. **A**, **B**: expanded polytetrafluoroethylene (ePTFE): AFX2 (Endologix Inc., Irvine, USA) after EndoAnchor (EA) removal. **C**–**E**: ePTFE: Excluder (W.L. Gore & Assoc., Flagstaff, USA) with an EA in place (**C**) and (**D**, **E**) with the EA removed. **F**, **G**: polyethylene terephthalate (PET) fabric: Endurant II (Medtronic Vascular, Santa Rosa, USA). **F**: a round hole after EA removal. The black arrow in image **G** points to the enlarged perforation due to the placement of an EA in contact with a metal stent. **H**: PET fabric: Zenith Renu (Cook Aortic Interventions, Bloomington, USA) endocuff with a round hole after EA removal. **I**–**N**: fluorescent microscopy, 4x/0.013–20x/0.5 zoom. **I**: an ePTFE perforation (AFX2). **J**, **K**: ePTFE perforation (Excluder) with dissection like alteration. **L**: PET perforation (Endurant II) with distorted and ruptured filaments. **M**, **N**: PET perforation (Renu) with distorted, partially broken and ruptured filaments.

After careful removal of the EAs, a detailed analysis revealed oval, smooth edged perforations in the ePTFE grafts (Fig. 1A–E) and round, smooth edged perforations in the PET grafts (Fig. 1F–H).

The holes in the AFX2 endoprosthesis could be characterised as apertures perforated straight through the material (Fig. 1A–B). Conversely, the perforations in the Excluder endocuff presented as multidirectional and multilayer tears. Although the perforation channel itself retained the form of an EA track, local dissection like damage of torn layers extended further (Fig. 1C–E). The perforations in the Endurant II and Renu endocuffs were similar, characterised by holes punctured straight through the material (Fig. 1F–H).

### High sensitivity fluorescent microscopy

The fluorescent microscopy of the perforations in the AFX2 cuff revealed folded contralateral edges of the perforations, with openings that had otherwise broken straight through the material (Fig. 1I). The perforations in the Excluder endocuff were characterised by multilayer dissections of the ePTFE material in different directions; the layers were thin and could be easily damaged (Fig. 1J and K). The holes in Endurant II and Renu endocuffs were similar straight through punctures. As a result of the introduction of EAs, multiple PET fibres were locally elongated, distorted, or ruptured (Fig. 1L–N).

### Surface area and perimeter of perforations

A summary of hole perimeters, perforations, and EA shaft cross sectional areas (based on the degree of penetration) is presented in Table 2. Owing to the endocuff material elasticity, the sizes of some perforations diminished within minutes after EA removal, which prevented hole measurements being obtained before the recoil. Compared with the EA shaft cross sectional area, the actual precise area of holes after EA removal was smaller for the PET EGs (180 000 μm^2^ [IQR 10 000] *vs*. 50 969 μm^2^ [IQR 57 161]; *p* = 0.008; Mann–Whitney *U* test). The recoil of the ePTFE perforations was insignificant (180 000 μm^2^ [IQR 0 μm^2^] *vs*. 164 408 μm^2^ [IQR 169 130 μm^2^]; *p* = 1.0; Mann–Whitney U test). The median recoil values for ePTFE and PET are presented in Table 3. A nearly statistically significant difference was observed, in that the perforations in the AFX2 endocuff were the largest in size (251 182 μm^2^ [IQR 667 062 μm^2^]) compared with the holes in other cuffs (97 625 μm^2^ [IQR 74 001 μm^2^], *p* = 0.050; Mann–Whitney *U* test). The detectable median perforation area and perimeter were satistically significantly larger in the ePTFE grafts compared with the PET fabric (Table 3).

**Table 2:** Post-removal characteristics of EndoAnchor perforations in different types of endocuff material.

Material	Hole number	Hole shape	CSAEA – μm^2^	Precise hole area – μm^2^	Hole perimeter – μm	Hole area per cuff – μm^2^	Hole perimeter per cuff – μm
ePTFE	1E	Oval	180 000	103 916.35	1 471.48		
ePTFE	2E	Oval	180 000	301 179.82	1 211.10	391 569.84 ±387 416.58	1 638.14 (3 645.00)
ePTFE	3E	Oval	180 000	201 183.18	1 804.79		
ePTFE	4E	Oval	180 000	960 000.00	5 960.00		
ePTFE	1G	Oval	180 000	206 322.15	1 828.11		
ePTFE	2G	Oval	180 000	127 633.51	1 405.54	137 611.61 ± 47 964.09	1 536.17 ± 205.71
ePTFE	3G	Oval	180 000	121 591.00	1 379.22		
ePTFE	4G	Oval	160 000	94 899.78	1 531.82		
PET	1M	Round	180 000	50 969.31	888.84	77 872.41 ± 38 046.72	1 061.63 ± -
PET	2M	Round	180 000	104 775.50	1 234.41		
PET	1C	Round	180 000	37 825.99	757.77		
PET	2C	Round	180 000	97 625.00	1 182.94	61 901.32 ± 31 555.40	931.25 ± 223.11
PET	3C	Round	160 000	50 252.97	853.04		

Data are shown as median (IQR) or mean ± SD.

Abbreviations: CSAEA = cross sectional area of an EndoAnchor shaft; ePTFE = expanded polytetrafluoroethylene; PET = polyethylene terephthalate; IQR = interquartile range; SD = standard deviation. 1E-4E = AFX2 (Endologix Inc., Irvine, USA); 1G-4G = Gore Excluder (W.L. Gore and Assoc., Flagstaff, USA); 1M-2M = Endurant II (Medtronic, Minneapolis, USA); 1C-3C = Zenith Renu (Cook Aortic Interventions, Bloomington, USA). Precise hole area = perforation area after the removal of an EndoAnchor.

**Table 3:** A summary of outcomes regarding material defect characteristics following EndoAnchor placement in the aortic endocuffs.

Parameter	ePTFE	PET	*p* value
Number of endocuffs studied	2	2	n/a
Number of EAs applied	8	5	n/a
EA penetration angle – °	80 ± 10	77 ± 8	0.56∗
Perforation shape	Oval	Round	n/a
Major material alterations	Large tears, layer dissections	Filament ruptures and elongations	n/a
Median hole area – μm^2^	164 408 (169 130)	50 969 (57 161)	0.011†
Median hole perimeter – μm	1 502 (436)	889 (403)	0.003†
Median material recoil after EA removal – μm^2^	15 591 (160 893)	109 747 (56 803)	0.003†

Data are shown as mean ± SD or median (IQR).

Abbreviations: ePTFE = expanded polytetrafluoroethylene; PET = polyethylene terephthalate; EA = EndoAnchor; EG = endograft; SD = standard deviation; IQR = interquartile range; n/a = not applicable.

∗ Independent samples t test.

† Mann–Whitney *U* test.

Three EAs were placed in contact with metal stent structures. Additional fabric tension created by an EA supported by a metal stent visually increased the perforation area (Fig. 1G). The perforations diminished in size once the EAs were carefully removed. No statistically significant difference was found between the area and perimeter of holes made by the EAs with or without contact with metal stent structures after device removal.

The penetration angles described in Table 1 had no significant impact on the area and perimeter of perforations. Two EAs were introduced at approximately one quarter of the device length. A slightly smaller diameter of an EA screw at this level had no significant impact on the differences of area and perimeter of perforations between the ePTFE and PET EGs.

## DISCUSSION

Endoanchors can create perforations that are different in shape and size. Their characteristics depend on the material through which they are passed. The defects caused by EAs in ePTFE material are significantly more extensive than those caused in PET.

A lack of sealing for various reasons, including complex anatomy,^9^^,^^21^ a misplaced EG, EG migration,^22^ aneurysm disease progression, and neck dilatation^13^ can potentially be corrected or compensated for by endosuturing or endostapling. According to the data of 221 patients from the PERU registry, the operational technical success was high (89%).^23^ The freedom from T1aEL post-ESAR was 94% at two years.^23^ According to Qamhawi et al., T1aEL occurs in 3.5% of all cases within 15 months post-procedure.^16^ Given the herein proven material recoil, particularly in the PET fabric, it is possible that filaments form a tight seal around the EA, which may increase EA stability and help reduce the risk of EL after successful EA implantation.

A previous explantation analysis of two EGs with a combined number of 13 EAs implanted for T1aEL revealed alterations in textile structure in the EA area.^24^ The precise analysis of perforations in the current series provides similar evidence, revealing multiple fibre ruptures and elongations in PET, as well as tears and layer dissections in ePTFE. In textile materials that are under continuous cardiac pressure load, filament alterations and breaks caused by an EA could lead to their consequent rupture, particularly in the absence of a properly apposed aortic wall, which would act as a buffer. Hypothetically, local contact pressure from an EA could further advance yarn separation and increase the size of a hole over time, especially in cases with complex anatomy and partial penetration of EAs at suboptimal angles.

The ePTFE membrane structures of the Excluder and AFX2 endocuffs are different. The main visible difference is the several layer design of the former. The spindle shaped perforations of the dissected Excluder ePTFE sheets open in different directions, which allows the underlying layers to partially cover the more superficially situated openings. The perforations made in the single layer ePTFE material of the AFX2 were straight through apertures; therefore, they were characterised by larger areas compared with the EA shaft cross sectional area. The use of EAs in conjunction with the Endologix Powerlink is contraindicated.^19^ Although the thinner membrane of the AFX2 is stronger and less permeable than the ePTFE of the first generation Powerlink,^25^ the materials of both products obviously share similarities and could therefore be prone to more extensive damage. It is believed that there are no other bench tests in the literature that take this issue into consideration, which adds value to this work. The impact of EA application on the other EGs was similar among the studied samples, which is in line with the general instructions for use.^19^ Nonetheless, available data on the long term outcomes of the introduction of an EA to EG fabric are insufficient. A recent report from the ANCHOR Registry is encouraging, even though the studied cohort included 70 primary treatment patients with short aortic necks with EAs applied into Medtronic Endurant EGs. Nine patients presented with T1aEL within five years.^12^ According to data from the current group, it appears that an EG can undergo further degradation once damaged, leading to device instability over time.^7^^,^^24^ Although complete replications of intravascular conditions seem difficult, an assessment of possible post-ESAR EG membrane degradation using machine fatigue tests *in vitro* is possible and would provide valuable information on the durability of EGs and reliability of ESAR.

This study had several limitations. First, this pilot experiment was performed on a small number of EGs, and although the conclusions may be based only on these samples, it would be reasonable to assume that, given the strict manufacturers’ quality control on endograft material, such results can possibly be extrapolated to devices from the companies represented above. Statistical analyses were limited for the same reason, and some of the results could be false negative. Second, continuous cardiac load could not be applied in this experimental study, but that would not be realistic without considering the EGs with simulated apposition to the aorta. Third, long term outcomes of material degradation in relation to EA implantation cannot currently be provided, again with the consideration that this would necessarily need to mimic EG to aortic wall apposition.

### Conclusion

During EA placement, the EG membrane undergoes local alteration and or destruction. In addition, ePTFE, particularly AFX2 endocuffs (for which EA use is not recommended), are characterised by a more extensive degree of material alteration compared with PET fabric. Additional longitudinal studies are required to confirm these findings in fatigue tests.

## CONFLICT OF INTEREST

None.

## FUNDING

None.
